# Case Report: Soft endoscopic treatment of Herlyn–Werner–Wunderlich syndrome: an innovative clinical procedure for hymen preservation

**DOI:** 10.3389/fmed.2026.1771221

**Published:** 2026-09-03

**Authors:** Yonghua Tian, Wei Fu, Yan Geng

**Affiliations:** 1 Department of Obstetrics and Gynecology, 923th Hospital of PLA Joint Logistics Support Force, Nanning, China; 2 Department of Gastroenterology, 925th Hospital of PLA Joint Logistics Support Force, Guiyang, China; 3 Department of Gastroenterology, 923th Hospital of PLA Joint Logistics Support Force, Nanning, China

**Keywords:** endoscopic septotomy, ESD, flexible gastroscope, Herlyn–Werner–Wunderlich syndrome, hymen-sparing, OHVIRA, virgin

## Abstract

**Background:**

Herlyn–Werner–Wunderlich syndrome (HWWS) management typically requires hymenotomy. We describe the fully endoscopic, hymen-sparing septotomy in a patient with no history of sexual intercourse.

**Case presentation:**

A 20-year-old patient with no prior sexual intercourse and progressive dysmenorrhea was diagnosed by MRI with HWWS, presenting as uterus didelphys, obstructed right hemivagina, and left renal agenesis. A 9-mm flexible gastroscope and endoscopic submucosal dissection (ESD) knives were introduced through the intact hymen to resect an 11 × 13 mm oblique septum, evacuating 20 mL of chocolate fluid. The hymen remained intact. The patient was discharged on postoperative day 3 and remained asymptomatic at 2-month follow-up, with no residual hematocolpos detected on ultrasound. Telephone follow-up at 12 and 18 months postoperatively confirmed no recurrence of dysmenorrhea.

**Conclusion:**

Endoscopic septotomy safely relieves HWWS obstruction while preserving hymenal integrity in patients with no sexual history. This novel technique addresses a crucial patient-centered need and expands therapeutic options for complex Müllerian anomalies.

## Introduction

Herlyn–Werner–Wunderlich syndrome (HWWS), also known as obstructed hemivagina and ipsilateral renal anomaly (OHVIRA) syndrome, represents a rare congenital Müllerian and mesonephric duct anomaly characterized by the classic triad of uterus didelphys, an obstructed hemivagina, and ipsilateral renal agenesis (1). This malformation typically remains asymptomatic until after menarche, when the accumulation of menstrual blood in the obstructed hemivagina leads to the formation of a hematocolpos, manifesting clinically as progressively worsening dysmenorrhea, a palpable paravaginal mass, and occasionally intermenstrual spotting or urinary symptoms (2, 3). The nonspecific nature of these symptoms, coupled with the variability in anatomical presentation, frequently results in diagnostic delays or misdiagnosis, which can precipitate a cascade of complications including recurrent urinary tract infections, endometriosis, pelvic inflammatory disease, and compromised future fertility (4–6).

Accurate and timely diagnosis is therefore paramount to mitigate these long-term sequelae. While no single imaging modality serves as an absolute gold standard, pelvic ultrasound and magnetic resonance imaging (MRI) are the cornerstones for delineating the complex pelvic anatomy, with MRI offering superior soft-tissue contrast for precise preoperative planning (6, 7). The definitive treatment for HWWS is surgery, aimed at relieving the outflow obstruction by resecting the vaginal septum. Early intervention is advocated to decompress the hematocolpos, alleviate symptoms, and prevent the development of endometriosis and adhesions that could adversely affect reproductive potential (8).

Conventional surgical approaches to vaginal septotomy, whether performed via a transvaginal or hysteroscopic route, often necessitate some degree of hymenal disruption to achieve adequate exposure for instrument access. This presents a significant clinical and ethical dilemma when managing patients with no sexual history for whom hymenal integrity may hold considerable cultural, religious, or personal importance. The lack of a well-described, minimally invasive technique that effectively resolves the anatomical obstruction while definitively preserving the hymen represents a notable gap in the therapeutic arsenal for this specific patient population. In this report, we describe, to our knowledge, the first case of HWWS-related oblique vaginal septum resection performed using a flexible gastroscope combined with ESD instruments through an intact hymenal orifice. Although ESD devices have previously been used in hysteroscopic surgery, the novelty of this approach lies in the hymen-sparing transhymenal use of a flexible gastrointestinal endoscope, which allows multidirectional manipulation within the narrow vaginal fornices and may provide a minimally invasive option for selected patients with complex obstructive Müllerian anomalies.

## Case presentation

### Patient information

A 20-year-old nulligravid woman with an intact hymen presented with a four-year history of progressively worsening dysmenorrhea since menarche at age 16. Her pain score on a visual analog scale (VAS) was 8/10 during menstruation, requiring regular nonsteroidal anti-inflammatory drugs with incomplete relief. She denied intermenstrual bleeding, urinary symptoms, or previous abdominal surgery. Family history was negative for congenital anomalies. Written informed consent was obtained from the patient for publication of this case report and any accompanying images.

### Clinical findings

Physical examination, performed with respect to her virginity, revealed normal external genitalia with an intact hymen. On bimanual rectal examination (performed with consent), a tense, tender paravaginal mass was palpable on the right side. Laboratory evaluation showed normal renal function: serum creatinine 0.68 mg/dL and estimated glomerular filtration rate (eGFR) 115 mL/min/1.73m^2^.

### Diagnostic assessment

Pelvic MRI with three-dimensional reconstruction confirmed the diagnosis of HWWS/OHVIRA syndrome. Key findings included.

Uterus didelphys with two separate uterine cavities and cervices.

Left renal agenesis; right kidney was normal in size and position.

**Figure 1:**
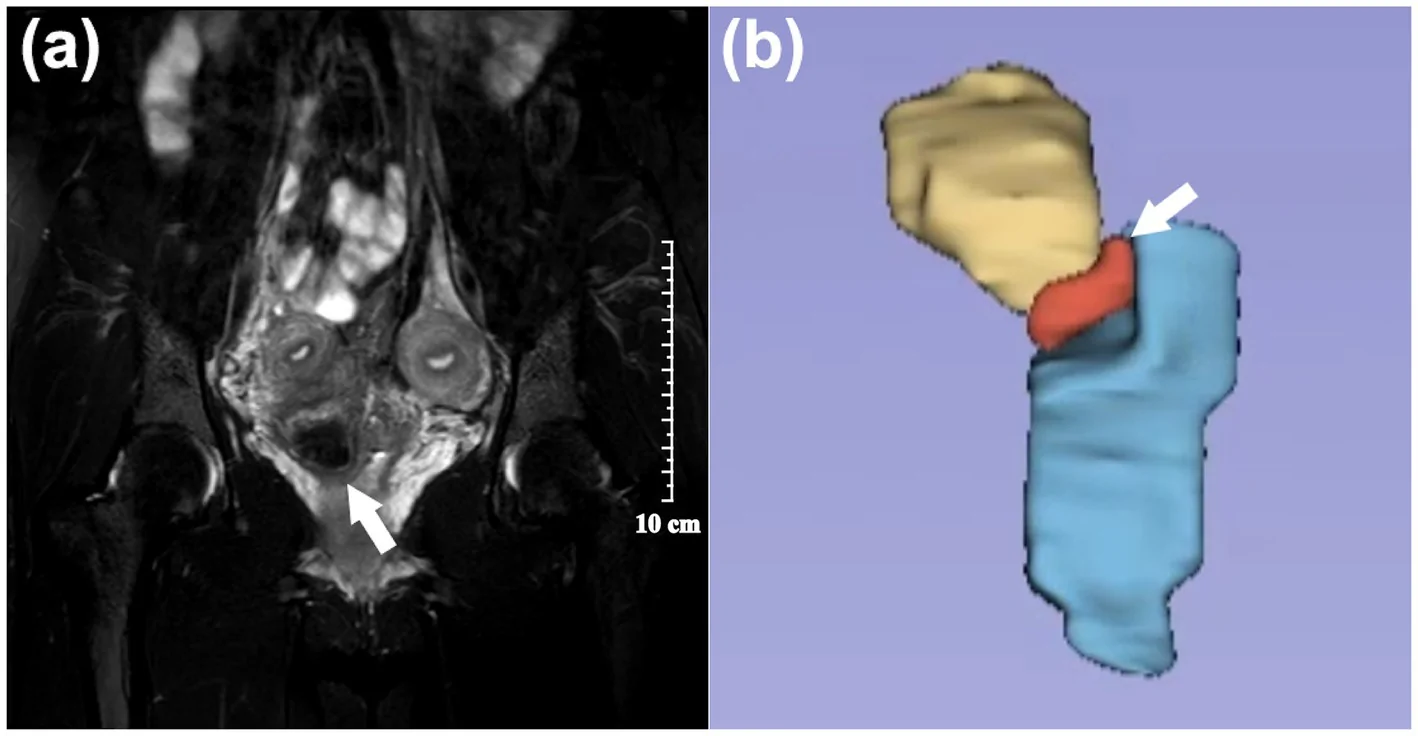
Preoperative MRI **(a)** Sagittal T2-weighted MRI showing uterus didelphys (U), obstructed right hemivagina with hematocolpos (H), and the oblique vaginal septum (arrow). The septum measured 11 mm in thickness and 13 mm in length. **(b)** Three-dimensional reconstruction demonstrating two separate uterine cervices (arrows) and left renal agenesis.

An oblique vaginal septum (thickness: 11 mm, length: 13 mm) obstructing the right hemivagina that led to a distended right vaginal cavity containing a hematocolpos measuring 5.2 × 4.1 × 3.8 cm, with a volume of 42.3 mL calculated using the formula length × width × height × 0.52 (Figure 1a).

Differential diagnoses considered and ruled out based on imaging and clinical presentation included transverse vaginal septum, imperforate hymen, cervical agenesis, and unicornuate uterus with a noncommunicating rudimentary horn. The diagnosis was confirmed in a multidisciplinary team meeting involving gynecologists, radiologists, and urologists.

### Therapeutic intervention

**Figure 2:**
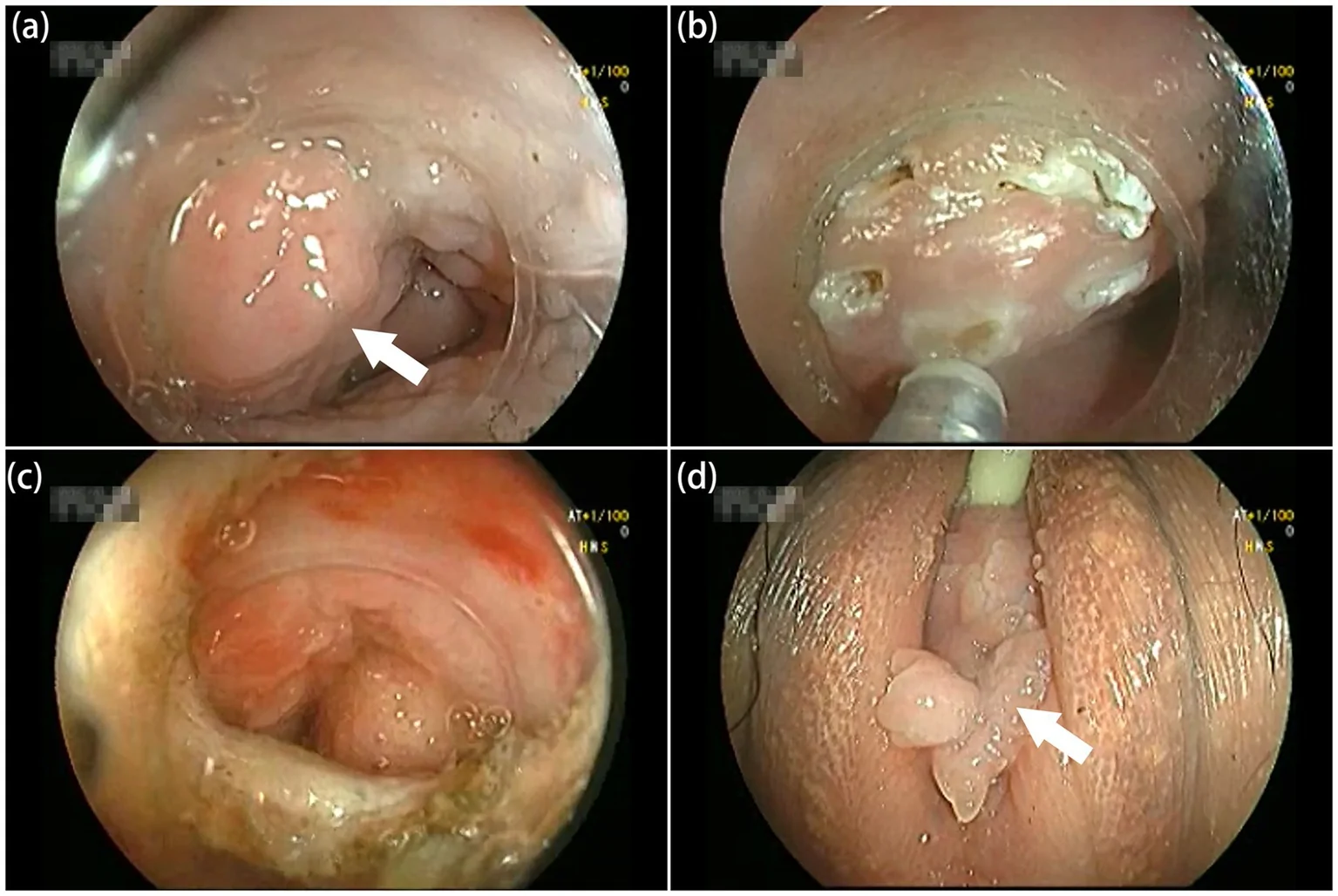
Intraoperative endoscopic views. **(a)** Vaginal septum bulging 1 cm below the left cervical margin (white arrow). **(b)** Resection margin marked at the same bulging site. **(c)** Incision afforded a clear view of the right vagina and cervix; neither was injured. **(d)** Endoscope removed without drainage; hymen remained intact.

Under propofol sedation, the patient was placed in a low lithotomy position. A 9-mm outer diameter flexible gastroscope (Fujinon BL-7000) was gently introduced through the hymenal orifice into the non-obstructed left vaginal canal. The procedure was performed using a standard single-operator gastrointestinal endoscopic technique, with fine knob adjustments to adapt the endoscope to the acute angle of the vaginal fornix. The left cervix was visualized, and an oblique vaginal septum measuring 11 × 13 mm was identified approximately 1 cm inferior to it (Figure 2a).

No submucosal injection was performed because the septum was fibrous and unsuitable for fluid lifting. Using a disposable Olympus DualKnife, the septum was incised layer by layer under direct visualization until the obstructed cavity was entered, releasing approximately 20 mL of dark, viscous retained blood. After complete aspiration, a disposable Olympus IT knife was used to resect the remaining septum along the contralateral vaginal wall. The electrosurgical settings were Endo Cut I (effect 3, cut duration 4, cut interval 4) for incision and Swift Coag (effect 2, 50 W) for hemostasis. No uterine distension medium was used.

**Figure 3:**
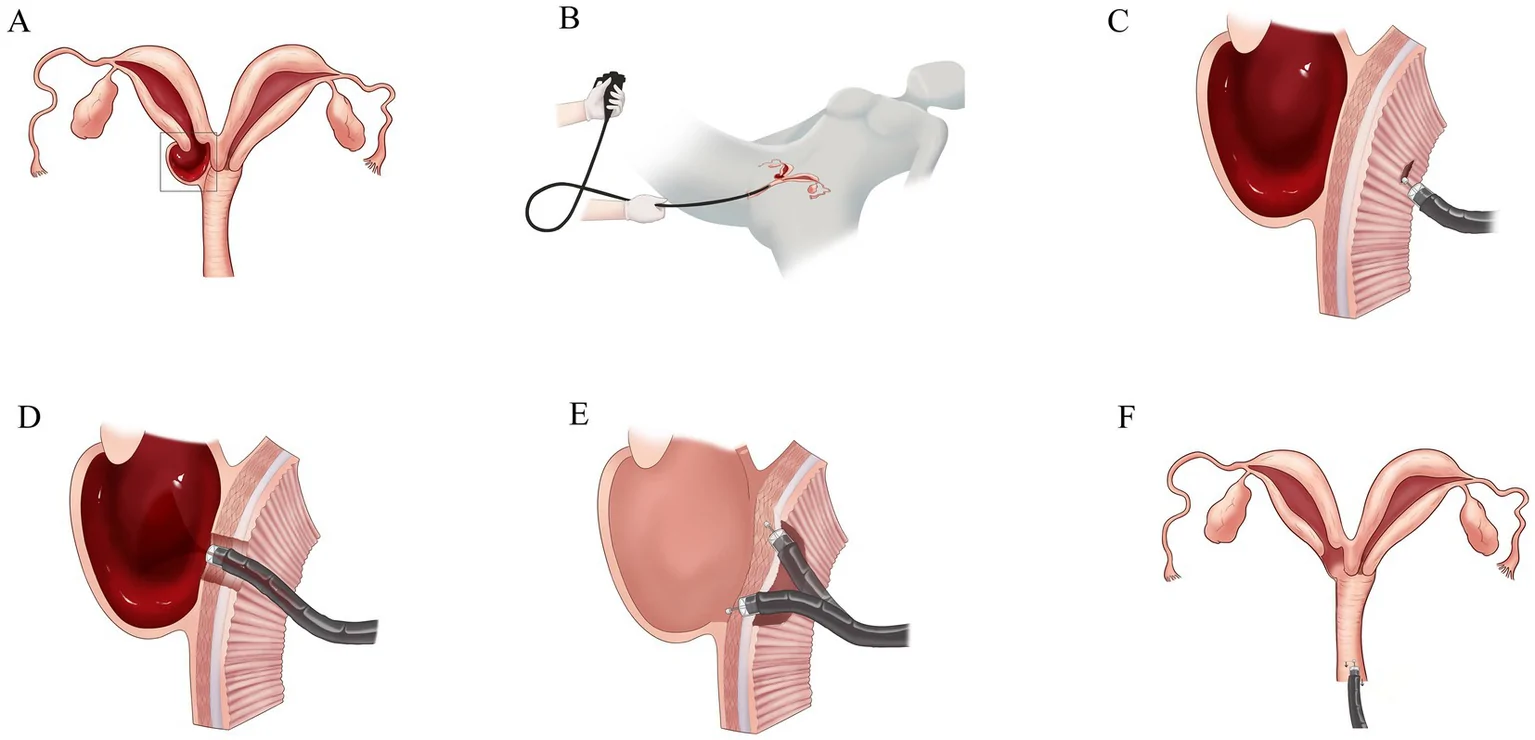
Stepwise flexible endoscopic transhymenal resection of an obstructing oblique vaginal septum in OHVIRA syndrome. **(A)** Uterus didelphys with an obstructed right hemivagina and hematocolpos caused by an oblique vaginal septum. **(B)** Introduction of a flexible gastroscope through the natural hymenal orifice into the patent left hemivagina, with preservation of hymenal integrity. **(C)** Endoscopic identification of the oblique vaginal septum and layer-by-layer incision using a DualKnife. **(D)** Entry into the obstructed right hemivaginal cavity, followed by drainage and aspiration of retained dark menstrual blood. **(E)** Resection of the residual vaginal septum using an IT knife, creating a common vaginal cavity and exposing the right cervical os. **(F)** Final anatomy after complete septal resection, demonstrating restored vaginal drainage and preservation of hymenal integrity.

The incision was extended to fully expose the right cervical os (Figure 2c). Complete resection was confirmed by visualization of both cervices and the newly created common vaginal cavity. Hemostasis was achieved with pinpoint coagulation using the IT knife; no sutures or vaginal packing were required. The total procedure time was 1 h and 38 min, and the estimated blood loss was less than 5 mL. The hymen remained intact throughout the procedure and upon withdrawal of the endoscope (Figure 2d). Figure 3 provides a schematic overview of the operative steps involved in the repair.

### Follow-up and outcomes

**Figure 4:**
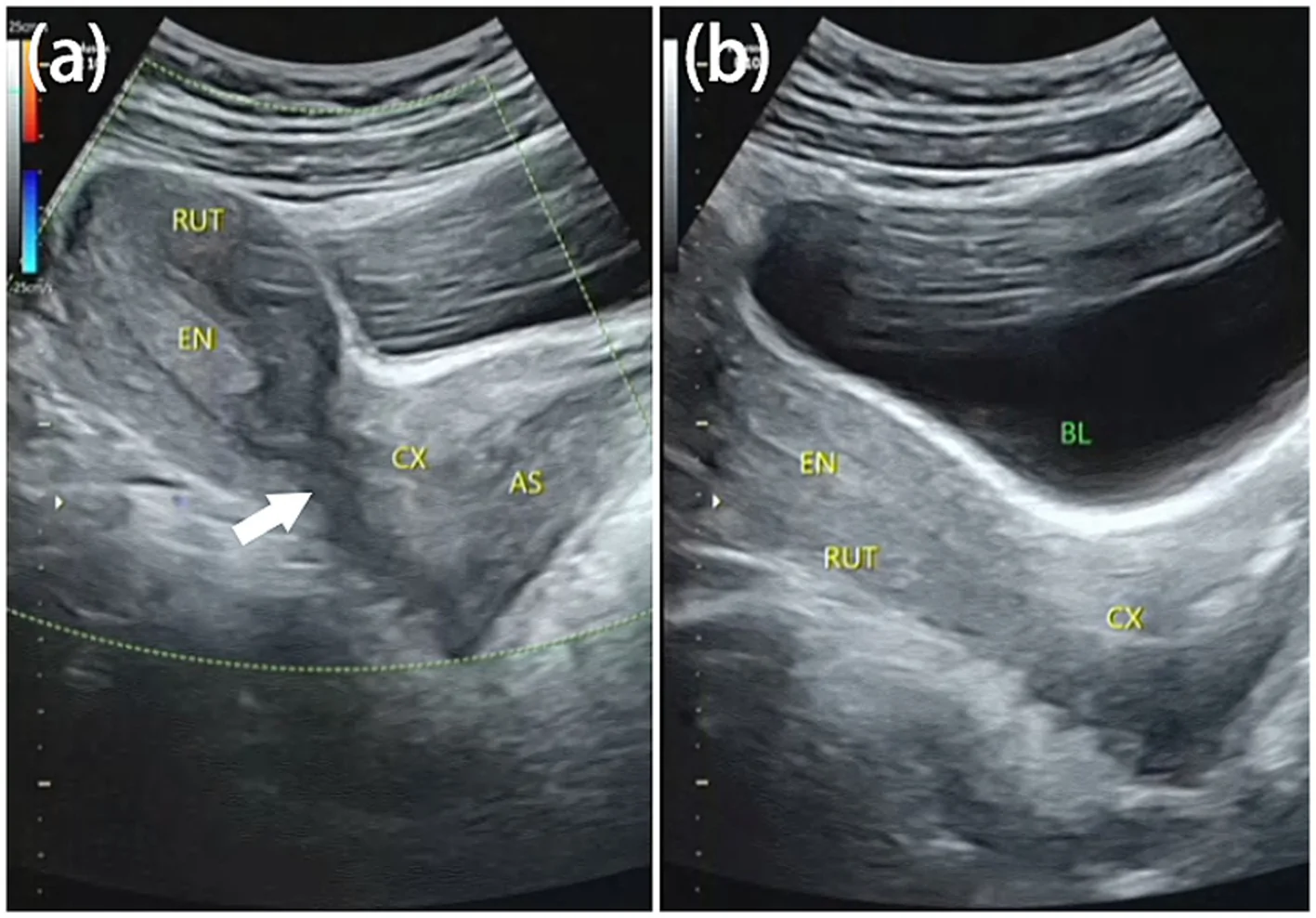
Ultrasound findings **(a)** Preoperative scan showed a fluid-filled dark area in the right vagina (hematocolpos, white arrow). **(b)** Post-menstruation scan (2 months) verified no residual hematocolpos or hematometra.

The patient was discharged on postoperative day 3 after an uneventful recovery. She received a 1-day course of intravenous antibiotics (cefuroxime) for prophylaxis. At the 2-month follow-up, she reported complete resolution of dysmenorrhea (VAS 0/10). During telephone follow-up at 12 and 18 months postoperatively, she reported no recurrence of dysmenorrhea. A post-menstrual transabdominal ultrasound showed no residual hematocolpos or hematometra, confirming patent drainage (Figure 4). No intraoperative or postoperative complications (bleeding, infection, or perforation) occurred.

### Clinical context

To our knowledge, the novelty of this case lies in the use of a flexible endoscopic technique performed via a hymen-sparing transhymenal approach for the treatment of HWWS. Martini et al. (9) previously described a case of a 17-year-old patient with HWWS and no sexual history managed by transvaginal septotomy with hymenal preservation in a challenging socio-cultural setting in Syria, but their technique still required a small vaginal incision. In contrast, our approach utilizes a flexible endoscope through the natural hymenal orifice, achieving complete hymenal preservation without any incision. Other minimally invasive techniques, such as in-office hysteroscopic treatment reported by Fascilla et al. (10), have demonstrated efficacy but do not specifically address or guarantee hymenal preservation. Our technique thus fills a distinct gap in the therapeutic arsenal for this specific patient population.

## Discussion

The conventional surgical management of HWWS has traditionally involved transvaginal septotomy, which typically necessitates hymenotomy to access the obstructed hemivagina. While effective in relieving obstruction, this approach inherently compromises hymenal integrity—a significant concern for patients with no sexual history due to cultural, religious, or personal reasons. The present case fundamentally diverges by employing a fully endoscopic approach using a flexible gastroscope and endoscopic submucosal dissection (ESD) instruments, allowing precise layer-by-layer dissection through the natural hymenal orifice without any incision. This case highlights the innovative use of flexible endoscopy via a hymen-sparing transhymenal approach for the treatment of HWWS. The flexibility of the 9-mm gastroscope enabled navigation of the acute forniceal angle—a technical challenge not encountered in upper gastrointestinal endoscopy (11, 12). The use of a HookKnife for meticulous dissection prevented premature collapse of the obstructed cavity, a detail critical for maintaining a clear operative field (13).

Compared to existing techniques, our approach offers several distinct advantages: (1) it is truly hymen-sparing, avoiding any incision or dilation of the introitus; (2) it provides excellent visualization and precise dissection using ESD principles borrowed from gastrointestinal endoscopy; (3) it eliminates the need for vaginal packing or sutures, facilitating rapid postoperative recovery; and (4) it can be performed without specialized gynecologic endoscopic equipment, making it potentially accessible to centers with therapeutic endoscopy expertise.

The successful execution of this technique hinges on several critical technical and diagnostic points. Foremost, precise preoperative anatomical delineation via magnetic resonance imaging (MRI) is indispensable, as it accurately maps the septum’s thickness, location, and spatial relationship to the cervix, thereby guiding the endoscopic trajectory and minimizing the risk of iatrogenic injury (14). This diagnostic precision is paramount, as delayed or missed diagnosis can lead to severe complications including endometriosis and infertility (15). The endoscopic technique itself offers distinct advantages. The layer-by-layer dissection using ESD instruments, a principle borrowed from gastrointestinal surgery, allows for controlled incision and minimizes bleeding, which is crucial in the confined vaginal space (16). This meticulous approach prevents the premature collapse of the obstructed hemivagina—a potential pitfall that could obscure visualization and complicate the resection—thereby ensuring complete septal removal and adequate drainage. Furthermore, the procedure’s minimally invasive nature, obviating the need for sutures or vaginal packing, contributes to rapid postoperative recovery and excellent cosmetic and functional outcomes by preserving the hymenal anatomy.

However, this technique is not without its limitations and requires careful patient selection. It is most suitable for patients with a well-defined, non-extremely thick septum and favorable anatomy accessible via the hymenal orifice. Cases involving severe vaginal adhesions, an exceptionally thick septum, or complex anatomical variations may still necessitate traditional transvaginal or combined laparoscopic approaches (17). In the present case, the Olympus HookKnife and IT knife were single-use devices, with additional costs mainly arising from disposable ESD instruments and endoscope use and disinfection. However, beyond hymenal preservation, the flexible endoscopic platform enables multidirectional cutting at variable angles and may be advantageous over hysteroscopic instruments in selected HWWS cases with a limited operative field, steep resection angles, or a relatively thick septum. The presented case also underscores the evolving paradigm towards individualized, patient-centered care in managing congenital anomalies, particularly for adolescents and young women where anatomical and psychosocial factors are equally significant (18).

As a single-center single case report, the findings cannot be generalized and the level of evidence remains low. Although the patient remained free of recurrent dysmenorrhea during 18 months of follow-up, longer follow-up beyond 3 years is still needed to evaluate outcomes such as septum re-adhesion, recurrence of obstruction, and potential effects on future fertility. In addition, this technique requires endoscopic submucosal dissection (ESD) experience and familiarity with flexible endoscopic manipulation in a narrow vaginal space. Whether laparoscopic backup is necessary should be further evaluated in a larger number of cases. The absence of a control group also precludes direct comparison with conventional surgical techniques regarding safety and efficacy. Based on this initial experience, preliminary selection criteria may include a vaginal canal that can accommodate a 9-mm endoscope and the absence of acute infection. Future studies with larger cohorts, prospective designs, and extended follow-up are essential to validate the long-term efficacy, safety, and reproductive impact of this endoscopic platform, as well as its potential applicability to other obstructive vaginal anomalies in pediatric and adolescent gynecology.

## Conclusion

This case demonstrates that fully endoscopic, hymen-sparing septotomy using a flexible gastroscope and ESD instruments is a feasible, safe, and effective technique for managing symptomatic HWWS in patients with no history of sexual intercourse. It successfully addresses the pathophysiological need for early obstruction relief while respecting the patient’s anatomical and personal integrity. This innovative approach represents a significant advancement in patient-centered care for this specific demographic and highlights the value of cross-disciplinary collaboration in developing novel surgical solutions.

## Data Availability

The raw data supporting the conclusions of this article will be made available by the authors, without undue reservation.

## References

[1] Vaidya P Agarwal P Vaidya A. Herlyn-Werner-Wunderlich syndrome: a case report. JNMA J Nepal Med Assoc. (2023) 61:283–6. doi: 10.31729/jnma.8096, 37203938 PMC10231529

[2] Habbash ZE. Herlyn–Werner–Wunderlich syndrome: a rare case report. Cureus. (2023) 15:e35003. doi: 10.7759/cureus.35003, 36938199 PMC10022840

[3] Kozłowski M Nowak K Boboryko D Kwiatkowski S Cymbaluk-Płoska A. Herlyn–Werner–Wunderlich syndrome: comparison of two cases. Int J Environ Res Public Health. (2020) 17:7173. doi: 10.3390/ijerph17197173, 33007989 PMC7579596

[4] Reyes-Martinez DA Galvis-Pabón JS Nieves-Cuervo GM Manrique-Hernández EF Hurtado-Ortiz A Licht-Ardila M. Herlyn Werner Wunderlich syndrome. Case report. Taiwan J Obstet Gynecol. (2025) 64:330–3. doi: 10.1016/j.tjog.2024.10.017, 40049820

[5] Mohd Adzlan F Mohd K Ahmad N Ramli R. OHVIRA syndrome: clinical implications of a delayed diagnosis. BMJ Case Rep. (2024) 17:e259861. doi: 10.1136/bcr-2024-259861, 38782440

[6] dos Santos Figueiredo JM Figueiredo J. Herlyn–Werner–Wunderlich (HWW) syndrome in an adolescent. Cureus. (2025) 17:e96788. doi: 10.7759/cureus.96788, 41399562 PMC12702527

[7] Horst W de Melo RC Theilacker G Schmitt B. Herlyn–Werner–Wunderlich syndrome: clinical considerations and management. BMJ Case Rep. (2021) 14:e239160. doi: 10.1136/bcr-2020-239160, 33664029 PMC7934712

[8] Tomecka P Jagodziński A Łuczak J Waszczuk Ł Murawski M. Diagnostic challenges of OHVIRA syndrome-a case report. J Clin Med. (2025) 15:190. doi: 10.3390/jcm15010190, 41517439 PMC12786447

[9] Martini N Alabdallah E Al-Ghotani B Alawad I Tawashi N. Successful management of Herlyn–Werner–Wunderlich syndrome in a 17-year-old virgin girl in the challenging socio-cultural-logistic setting of Syria: a case report. Ann Med Surg (Lond). (2023) 85:1223–6. doi: 10.1097/MS9.0000000000000510, 37113823 PMC10129165

[10] Fascilla FD Olivieri C Cannone R de Palma D Manosperta F Costantino AS . In-office hysteroscopic treatment of Herlyn-Werner-Wunderlich syndrome: a case series. J Minim Invasive Gynecol. (2020) 27:1640–5. doi: 10.1016/j.jmig.2020.04.013, 32320799

[11] Repici A Spadaccini M Belletrutti PJ Galtieri PA Fugazza A Anderloni A . Peroral endoscopic septotomy for short-septum Zenker's diverticulum. Endoscopy. (2020) 52:563–8. doi: 10.1055/a-1127-3304, 32185781

[12] Zeng X Bai S Zhang Y Ye L Yuan X Hu B. Peroral endoscopic myotomy for the treatment of esophageal diverticulum: an experience in China. Surg Endosc. (2021) 35:1990–6. doi: 10.1007/s00464-020-07593-6, 32347387

[13] Kulkarni A Ruikar PD Alahabade R Mahajan R Kulkarni AV. Vaginoscopic resection of oblique vaginal septum in OHVIRA syndrome before menarche. J Minim Invasive Gynecol. (2023) 30:262–3. doi: 10.1016/j.jmig.2023.01.019, 36754275

[14] Choridah L Pangastuti N. Obstructed hemivagina and ipsilateral renal anomaly syndrome in an association with endometriosis: role of magnetic resonance imaging in diagnosis. Med J Malaysia. (2024) 79:83–6. 39215421

[15] Dahal P Tamang OY Upadhyaya RP Dawadi K Pradhan P Parajuli S. Herlyn–Werner–Wunderlich syndrome in a young female presenting with dysmenorrhea: a case report. Radiol Case Rep. (2024) 19:82–8. doi: 10.1016/j.radcr.2023.09.030, 37920694 PMC10618621

[16] Moneir WM Almodhen F Almaary J Almatar Z Alaqeel A. Vaginoscopic incision of vaginal septum with preservation of the hymen in a child with obstructed hemi-vagina ipsilateral renal agenesis (OHVIRA) syndrome. Cureus. (2022) 14:e30450. doi: 10.7759/cureus.30450, 36407203 PMC9672824

[17] Roziana R Nora H Maharani CR Yeni CM Dewi TP Rusnaidi R . Herlyn–Werner–Wunderlich syndrome: Challenges in diagnosis and management. Narra J. (2023) 3:e223. doi: 10.52225/narra.v3i2.223, 38450268 PMC10914055

[18] Oishi T Konishi K Okamoto M Mizoguchi C Yano M Kawano Y . Different interventions for OHVIRA/Herlyn-Werner–Wunderlich syndrome based on age and symptoms: a single-institution cohort study. BMC Womens Health. (2025) 25:350. doi: 10.1186/s12905-025-03905-x, 40671062 PMC12265284

